# Progress in Bacteriology

**Published:** 1904-04-30

**Authors:** 


### Progress in Bacteriology.

**Malaria.**—Mary E. Rowley [1] states that in æstivo-autumnal malaria in addition to the ring shaped bodies, crescents, and ovoids, there are to be found two other varieties of parasites. One is an intracorpuscular, elongated, sausage-shaped parasite with openings at the extremities, scattered pigment which is sometimes grouped at the end, and with dotted chromatin parallel to the long axis. The other a form of parasite intermediate between the crescent and the foregoing; curved, with the chromatin in dots at one end, and the pigment at the periphery. She inclines to the belief that these sausage-shaped bodies are developed from ring forms and are on their way to form crescents. Leishman [2] has recently demonstrated that important diagnostic points between the various types of malaria are brought out by Romanowsky's stain, especially if deep staining be employed. Thus, staining of moderate intensity in cases of tertian fever brought out Schüffner's dots in the red cells invaded by the parasite and this change was characteristic of a tertian invasion. Quartan fever did not affect the red cells in any such way. Malignant cases, he thought, might possibly be due to invasion by two varieties of parasites, one exciting malignant quotidian fever and the other exciting malignant tertian fever. Certainly the results obtained by staining were contradictory, for whilst it was often possible to demonstrate the dots in the red cells infected by young malignant rhizouts, and deep-stained capsules surrounding the mature gametes or crescents, yet in other cases no such changes could be detected although the same staining methods were employed. Leishman calls attention to forms which seem to indicate that crescents may be derived from a circular intra-corpuscular parasite similar to the gametes of benign tertian and quartan malaria.

**Rheumatoid Arthritis.**—Poynton and Paine [3] have brought forward evidence to show that the intravenous injection of a diplococcus obtained from a joint affected by rheumatoid arthritis may excite in rabbits not only a multiple arthritis but a monaticular osteo-arthritis. In a later paper [4] they state that the organism is indistinguishable from the diplococcus rheumaticus, and that from a second case they were able to excite in rabbits lesions which were both rheumatic and rheumatoid in character. Hale White [5] has put on record an acute case of osteoarthritis in which a diplococcus was isolated from the knee-joint, but he does not regard it as identical with the diplococcus rheumaticus. Von Dungern and Schneider cultivated a diplococcus from a case of rheumatoid arthritis, and cultures inoculated into rabbits produced typical joint lesions; but, as Hale White points out, their results are discounted by the fact that the case had recently suffered from rheumatic fever. In White's case in addition to the diplococcus which was found in cultures made from a scraping of an affected knee-joint, there was also found in an enlarged mesenteric gland a diplobacillus, but neither of these organisms were pathogenic to animals. The diplococcus did not form chains, grew well on the ordinary media, produced acid but not gas, did not coagulate milk, and was a facultative anærobe. It stained by Gram's method.

**Acute Rheumatism.**—Meyer,[6] whose previous work on the tonsillitis of acute rheumatism confirmed the theory of a specific diplococcus as described by Triboulet, has made a further investigation into this matter. He failed in many attempts to isolate the diplococcus from the blood or joint fluid, but was successful in obtaining it from swabbings of the tonsils of 26 cases of rheumatic angina. Inoculated into rabbits the diplo-streptococcus gave rise to conditions of arthritis and endocarditis. There are, undoubtedly, great difficulties in isolating the organism from the blood, joint fluids, or urine. McCrae [7] failed to obtain it in 270 consecutive cases investigated at the Johns Hopkins Hospital, and Phillipp [8] failed in 30 cases at Prague. Sahli found a coccus morphologically resembling the staphylococcus citreus. He regarded the joint lesions as being due to multiple local infection by the bacteria. Schmidt [9] has tried Menzer's anti-rheumatic serum in three cases of acute rheumatic fever, eight of subacute and four of chronic rheumatism. He injected 15 to 20 c.cms. in the neighbourhood of the inflamed joint, and was of opinion that benefit was obtained especially in subacute cases which did not yield to ordinary remedies. The reaction with redness, swelling, and erythema he believes to be beneficial. Poynton [10] summarises Menzers' serum treatment of rheumatic fever as follows: The serum which is anti-bacterial is obtained from cultures made from the throats of persons suffering from rheumatic angina. The streptococcus so obtained is not however in Menzer's opinion a specific organism of rheumatic fever. The reaction in the joints sets in four to six hours after the injection with increase of the cardiac symptoms, swelling of the lymph glands and tonsillitis. The temperature falls by lysis and cure results from the increase of natural resistance that has been induced. The total amount of serum injected varies from 100 to 200 ccs. in doses of 10 to 15 ccs. at a time. It must be used guardedly in cases of high fever, pericarditis and pleurisy. Poynton says that he has himself tried an antistreptococcus serum in various rheumatic affections with disappointing results. Merck[11] gives the following details about Menzer's serum. It is a bacteriolytic serum prepared with the aid of living cultures of streptococci taken direct from human beings without passage through animals. The disintegration of the infecting streptococci is accompanied by a rise of temperature and local painful swelling of the affected joints. The serum is injected subcutaneously in the thigh. In acute cases the maximum daily dose is 10 cc., and in all cases occasional intermissions should be made. Five to ten injections may be given. The serum may be given even if endocarditis be present, but caution is necessary if there be much pleural or pericardial exudation.

**Capsule Formation by Diplococcus Pneumoniæ in Culture.**—For the identification of the pneumococcus and to distinguish it from other diplo- or streptococci it is necessary either to stain films of the morbid material direct, or to inject the material into a mouse and examine the blood or exudate for encapsuled organisms after death. It is seldom possible to find capsules in organisms grown on culture media, even on media containing tracheal mucus or in milk as recommended by Washbourne. Gordon[12] has, however, found a method of obtaining cultures with capsules, and his procedure is as follows:—Add 1 litre of distilled water to 1 lb. of minced beef and boil for 30 minutes. Filter, add 12 per cent. of yellow gold table gelatine, 1 per cent. peptone, and ½ per cent. salt. Make faintly alkaline with liquor potassæ (B. P.). Add white of egg and steam for 30 minutes. Filter, pour into tubes, and sterilise for 30 minutes on two successive days. The material to be examined is inoculated upon the tube and incubated for 24 hours at 37° C. The liquid gelatine culture becomes turbid and a little is removed on a loop and spread on a cover-glass. The cover-glass is then dried over the flame and placed in alcohol for one minute, and then placed film downwards in carbol fuchsin for from one to three minutes. Finally, the film is rapidly immersed in water and mounted. Prepared in this way, capsules may be demonstrated around a good number of the diplococci. No such capsules can be demonstrated around streptococci prepared in the same way.

[1] Bull. Johns Hopk. Hosp, Jan., 1904. [2] Brit. Med. Jour., March 19. 1904. [3] Path. Soc. Transac., 1902. [4] Clin. Soc. Transac., Vol. XXXV. [5] Guy's Hosp. Rep., Vol. LVII. [6] Zeit. f. Klin. Med., Vol. XLVI., Nos. 5 and 6. [7] Jour. Amer. Med. Assoc., Jan., 1903. [8] Dent. Arch. fur Klin. Med., LXXVI., 1–3, 1903. [9] Munch. Med. Woch., XXXIX., 1903. [10] Practit., July, 1903. [11] Ann. Rep., Vol. XVI., May, 1903. [12] Brit. Med. Jour., March 26, 1904.

